# The value of bioethical research: A qualitative literature analysis of researchers’ statements

**DOI:** 10.1371/journal.pone.0220438

**Published:** 2019-07-29

**Authors:** Marcel Mertz, Tobias Fischer, Sabine Salloch

**Affiliations:** 1 Institute of History, Ethics and Philosophy of Medicine, Hannover Medical School, Hannover, Germany; 2 Institute of Ethics and History of Medicine, University Medicine Greifswald, Greifswald, Germany; 3 Clinic for Dermatology and Venereology, University Medical Center Rostock, Rostock, Germany; Utrecht University Medical Center, NETHERLANDS

## Abstract

**Introduction:**

Value and waste in preclinical and clinical research projects are intensively debated in biomedicine at present. Such different aspects as the need for setting objectives and priorities, improving study design, quality of reporting, and problematic incentives of the academic reward system are addressed. While this debate is also fueled by ethical considerations and thus informed by bioethical research, up to now, the field of bioethics lacks a similar extensive debate. Nonetheless, bioethical research should not go unquestioned regarding its scientific or social value. What exactly constitutes the value of bioethical research, however, remains widely unclear so far.

**Methods:**

This explorative study investigated possible value dimensions for bioethical research by conducting a qualitative literature analysis of researchers’ statements about the value of their studies. 40 bioethics articles published 2015 in four relevant journals (*The American Journal of Bioethics*, *Bioethics*, *BMC Medical Ethics* and *Journal of Medical Ethics*) were analyzed. The value dimensions of “advancing knowledge” (e.g. research results that are relevant for science itself and for further research) and “application” (e.g. increasing applicability of research results in practice) were used as main deductive categories for the analysis. Further subcategories were inductively generated.

**Results:**

The analysis resulted in 62 subcategories representing a wide range of value dimensions for bioethical research. Of these, 45 were subcategories of “advancing knowledge” and 17 of “application”. In 21 articles, no value dimensions related to “application” was found; the remaining 19 articles mentioned “advancing knowledge” as well as “application”. The value dimensions related to “advancing knowledge” were, in general, more fine-grained.

**Conclusions:**

Even though limitations arise regarding the sample, the study revealed a plethora of value dimensions that can inform further debates about what makes bioethical research valuable for science and society. Besides theoretical reflections on the value of bioethics more meta-research in bioethics is needed.

## Introduction

Bioethics as an academic field has emerged impressively since the 1970s and is characterized by its highly interdisciplinary nature and the need to integrate normative expertise with empirical data and practical experience [1, 2]. Having its origins in theology, moral philosophy, law as well as in the professional norms and standards of medical practice (e.g.[3]), the self-ascribed task of the field lies in questions of “What shall we (ethically) do?”–regarding the current, especially technology-driven, challenges in different subfields related to medicine, health care, public health, biotechnology, and biomedical research. More concretely, there is the expectation that bioethics will reflect upon ethical challenges and problems, analyze the norms and values involved or relevant, evaluate different courses of action or states of affairs, and propose viable solutions.

It is fair to assume at first that bioethics, understood in such a way, should have an impact on science and society—a sentiment probably shared within the community of bioethicists themselves, though contesting views are also aired, e.g. defending also the value of high-quality arguments even if they are not popular or result in changes in practice [4] or generally stressing the merits of “basic research” also in bioethics [5]. Other authors criticize the emphasis on “cashing out” research results in practice or policy changes as being a consequence of the fact that bioethics institutes are often “housed in academic medical schools rather than in traditional liberal arts universities” where the “academic currency gained from the bioethics discipline’s own criteria for the generation of new knowledge must always be *exchanged* for the currency of the professional school in order for bioethicists to pay their rent” ([6]; emphasis in the original). Notwithstanding these critical arguments, because bioethics is an *ethical* endeavor, it could *prima facie* be assumed that the respective research necessarily has an ethical and practical value. Reflecting on the increase of bioethical research and teaching in the last two or three decades, the professionalization and setting up of new institutions, and the growing number of research projects, a commitment to *some* valuableness of bioethical research is apparently widespread enough in Western societies to let their respective governments invest the necessary resources.

The question of “value and waste” is currently being debated intensively in the field of biomedical research [7, 8]) with respect to basic/preclinical and applied/clinical research projects [9, 10, 11, 12, 13]. There is a perceived need for a better research practice which extends to such different aspects as setting objectives and priorities, study design and monitoring, reporting and dissemination, and the academic reward system [8, 14, 15, 16]. The failure to secure mechanisms which lead to a better quality of research output is discussed worldwide and throughout various biomedical disciplines and branches. Starting from these comprehensive debates on the value and waste of biomedical research, one might feel invited to reframe the question of valuableness (and waste) with respect to bio*ethical* research. It could be argued that if something does not provide any value, it contributes necessarily to the waste of scientific and social resources. Critically questioning the value of bioethical research or making the value more transparent and explicit thus is indispensable for detecting waste in research.

In analogy with biomedical sciences the value of bioethical research must be critically examined regarding those aims which can be reasonably expected from this specific kind of investigation. In particular, it cannot be taken for granted that just because research is addressing ethical aspects, implications or arguments, it automatically provides valuable results for science and society. In fact, there can be several reasons why the value of bioethical research itself becomes questionable. Even though ethics deals with fundamental questions of good and bad, right and wrong or just and unjust, ethicists, as experts of normative questions, might fail regarding the value of their own research. For example, they can be biased concerning the relevance of the topics chosen or the practicability of a suggested solution. In addition, bioethicists are—like any other researchers—in danger of addressing predominantly those questions which are (i) of personal interest to them, (ii) help to promote their career (e.g. regarding publication and funding) or (iii) increase the power and/or reputation of their institutions or organizations, or of the field as a whole (e.g. increasing the influence of scientific or political agendas and establishing additional academic positions). As a special aspect, bioethical researchers may face the danger of confirming their own ethical convictions as a result of their research. Lastly, bioethics runs into danger to be used as a mere fig leaf in politics and for health care organizations, which is another factor that may reduce the value associated with bioethical research.

Whereas there is an ongoing intensive discussion about the value (and waste) of biomedical research, a debate of similar proportions regarding bioethics is yet lacking. There are contributions to the topic, for example the proposed conceptual model of bioethical research from Mathews and colleagues [17], albeit these are more directed to research *goals*, the problems of measurement of goal attainment, and different pathways to “translation” of bioethical research and scholarship. Furthermore, there are several attempts to strengthen the *quality* of bioethical methodology, for example, regarding applied philosophical bioethics [18], empirical-ethical research [19, 20] or (un-)systematic reviews, both empirical and reason-based [21, 22, 23, 24]. In addition, more practice-oriented aspects of the quality of bioethics are being currently discussed, for example, concerning Clinical Ethics Support Services [25, 26, 27], ELSI (ethical, legal and social issues) research [28], or ethics in HTA (health technology assessment) [29, 30]. Besides quality, however, also the “social value” of research—as vague the term often is [31]—can be of importance, as social value could be low even when quality is sufficient. Notwithstanding, a more general analysis of the value dimensions of bioethical research is still outstanding and would contribute to an overall estimation of the impact of bioethics on the advancement of research and on societal welfare.

So far, there has been no explicit framework developed for determining the value of bioethical research, although e.g. Hofmann and Magelssen descriptively listed some possible criteria recently based on a literature search on what constitutes “good bioethics” [18], which resulted in items such as “resulting in better health and wellbeing”, “resulting in changes in practice”, “legitimizing governance practices”, “opposing and correcting law” or “contribute to effective policy design” (see subheading “pragmatic concerns” in Table 1 of [18]). But fair and explicit measurement criteria are required to assess the value of a particular research project. A possible first strategy could be to draw an analogy to the “value and waste” debate in biomedicine and take the criteria discussed in this debate as a springboard for determining criteria for bioethical research. However, value dimensions of bioethical research may differ considerably from what is described for biomedical research and it can be reasonably debated whether bioethical research must be expected to have scientific and/or social value in a comparable sense. Preliminary work is thus needed to map the field regarding the distinct character of bioethics.

**Table 1:** List of the articles included in the analysis.

Authors	Title	Reference
**Journal: Bioethics**		
Carina Fourie	Moral distress and moral conflict in clinical ethics	[33]
Guy Kahane, Julian Savulescu	Normal human variation: refocussing the enhancement debate	[34]
Neil C. Manson	Transitional paternalism: How shared normative powers give rise to the asymmetry of adolescent consent and refusal	[35]
Andrew Mcgee	Acting to let someone die	[36]
Jeesoo Nam	Biomedical enhancements as justice	[37]
Vida Panitch	Assisted reproduction and distributive justice	[38]
Sandra K. Prucka, Lester J. Arnold, John E. Brandt, Sandra Gilardi, Lea C. Harty, Feng Hong et al.	An update to returning genetic research results to individuals: perspectives of the industry pharmacogenomics working group	[39]
Scott Brian Saxman	Ethical considerations for outcome-adaptive trial designs: a clinical researcher´s perspective	[40]
Nicolas Tavaglione, Angela K. Martin, Nathalie Mezger, Sophie Durieux-Paillard, Anne Francois, Yves Jackson et al.	Fleshing out vulnerability	[41]
Christopher Wareham, Cecilia Nardini	Policy on synthetic biology: deliberation, probability, and the precautionary paradox	[42]
**Journal: BMC Medical Ethics**		
Uría Guevara-López, Myriam M. Altamirano-Bustamante, Carlos Viesca-Trevino	New frontiers in the future of palliative care: real-world bioethical dilemmas and axiology of clinical practice	[43]
Irma M. Hein, Pieter W. Troost, Alice Broersma, Martine C. de Vries, Joost G. Daams, Ramón J.L. Lindauer	Why is it hard to make progress in assessing children´s decision-making competence?	[44]
Carly Hohm, Jeremy Snyder	"It was the best decision of my life": a thematic content analysis of former medical tourists´ patient testimonials	[45]
Tapani Keränen, Arja Halkoaho, Emmi Itkonen, Anna-Maija Pietilä	Placebo-controlled clinical trials: how trial documents justify the use of randomisation and placebo	[46]
**Pauline S.C. Kouwenhoven, Natasja J.H. Raijmakers, Johannes J.M. van Delden, Judith A.C. Rietjens, Donald G. van Tol, Suzanne van de Vathorst et al.**	Opinions about euthanasia and advanced dementia: a qualitative study among Dutch physicians and members of the general public	[47]
Mireille Lavoie, Gaston Godin, Lydi-Anne Vézina-Im, Danielle Blondeau, Isabelle Martineau, Louis Roy	Psychosocial determinants of physicians´ intention to practice euthanasia in palliative care	[48]
Bert Molewijk, Marit Helene Hem, Reidar Pedersen	Dealing with ethical challenges: a focus group study with professionals in mental health care	[49]
**Anke J.M. Oerlemans, Nelleke van Sluisveld, Eric S.J. van Leeuwen, Hub Wollersheim, Wim JM Dekkers, Marieke Zegers**	Ethical problems in intensive care unit admission and discharge decisions: a qualitative study among physicians and nurses in the Netherlands	[50]
Corinna Porteri, Carlo Petrini	Research involving subjects with Alzheimer´s disease in Italy: the possible role of family members	[51]
Ciara Staunton	Informed consent for HIV cure research in South Africa: issues to consider	[52]
**Journal: Journal of Medical Ethics**		
Andreas Albertsen, Carl Knight	A framework for luck egalitarianism in health and healthcare	[53]
Andreas Albertsen	Feiring´s concept of forward-looking responsibility: a dead end for responsibility in healthcare	[54]
Niklas Juth, Niels Lynöe	Zero tolerance against patriarchal norms? A cross-sectional study of Swedish physicians´ attitudes towards young females requesting virginity certificates or hymen restoration	[55]
Celia Kitzinger, Jenny Kitzinger	Withdrawing artificial nutrition and hydration from minimally conscious and vegetative patients: family perspectives	[56]
Joe Scott Mellor, Sally-Anne Hulton, Heather Draper	Adherence in paediatric renal failure and dialysis: an ethical analysis of nurses’ attitudes and reported practice	[57]
Francesca Minerva	Conscientious objection in Italy	[58]
**Natasja J.H. Raijmakers, Agnes van der Heide, Pauline S.C. Kouwenhoven, Ghislaine J.M.W. van Thiel, Johannes J.M. van Delden, Judith A.C. Rietjens**	Assistance in dying for older people without a serious medical condition who have a wish to die: a national cross-sectional survey	[59]
Ben Sauders	Is procreative beneficence obligatory?	[60]
Merle Spriggs, Lynn Gillam	Deception of children in research	[61]
M.A. Verkerk, Hilde Lindemann, Janice McLaughlin, Jackie Leach Scully, Ulrik Kihlbom, Jamie Nelson, Jacqueline Chin	Where families and healthcare meet	[62]
**Journal: The American Journal of Bioethics**		
**Katrien Devolder**	U.S. complicity and Japan´s wartime medical atrocities: Time for a Response	[63]
Autumn Fiester	Neglected ends: clinical ethics consultation and the prospects for closure	[64]
Jeremy R. Garrett	Collectivizing rescue obligations in bioethics	[65]
Allan J. Jacobs, Kavita Shah Arora	Ritual male infant circumcision and human rights	[66]
Liza-Marie Johnson, Christopher L. Church, Monika Metzger, Justin N. Baker	Ethics consultation in pediatrics: long-experience from a pediatric oncology center	[67]
Jennie Louise, Jaklin Eliott, Ian Olver, Anette J. Braunack-Mayer	Mandatory cancer risk warning on alcoholic beverages: What are the ethical issues?	[68]
Aasim Padela, Afshan Mohiuddin	Ethical obligations and clinical goals in End-of-life: deriving a quality-of-life construct based on the Islamic concept of accountability before God (taklif)	[69]
Adina Preda, Kristin Voigt	The social determinants of health: why should we care?	[70]
Robert Sparrow	Imposing genetic diversity	[71]
Anita J. Tarzian, Lucia D. Wocial, The ASBH Clinical Ethics Consultion Affairs Committee	A code of ethics for health care ethics consultants: journey to the present and implications for the field	[72]

### Research question and approach

The research question underlying this study can is therefore *What value dimensions do researchers in bioethical research address when reporting their research results*? We decided to apply a mixed strategy, not least because of the exploratory character of the research question. The methodology is based on theoretical (“deductive”) reflections about what generally constitutes “valuableness” in research, but is subsequently amended by a qualitative analysis of statements about the self-ascribed value of published bioethical research. The study has a “proof of concept” character in applying a qualitative approach to authors’ statements in journal articles as one source of information about value dimensions of bioethical scholarship.

The aim of the present study is, therefore, neither to determine the value of concrete bioethical projects nor to deal with the impact of bioethical scholarship which assesses the value of *biomedical* research, for example, through evaluation of projects in research ethics committees. In addition, this contribution will confine itself to the “value” aspect of the debate and does not aim to identify possible “waste” in bioethical research. In mapping the field, this contribution can be understood as a first preparatory step towards defining and operationalizing criteria which would finally allow the measurement of the value of particular bioethical research projects.

## Method

### Data sources

We decided to collect research articles from relevant publication organs in the fields of bioethics and medical ethics as sources for statements about the value of research in bioethics. The journals were selected using a list based on Harzing’s “Publish or Perish” rating [32], excluding those journals which have a specific focus other than general bioethics (e.g. on medical law or neuroscience). The following four journals were selected: *The American Journal of Bioethics* (Target Articles only), *Bioethics*, *BMC Medical Ethics* and the *Journal of Medical Ethics*. Regarding their relevancy, these four journals also corresponded to our own experience as researchers who publish and read papers in the field. There are of course also other important bioethical journals (also in other languages than in English), and a considerable amount of bioethical work is published in non-bioethical journals (e.g. medical or natural sciences journals). The restriction to four journals was, however, necessary to limit the quantity of articles undergoing qualitative analysis.

We then retrieved the first ten original articles published in each journal in 2015 (see Table 1). We excluded commentaries, letters and special issues. Apart from that, no other criteria for selecting articles were applied to uphold some randomness in the sample.

### Data extraction

The identification of relevant text passages was guided by the two main value dimensions suggested by Chalmers et al. [7]. Chalmers et al. understand *Advancing Knowledge* mainly in terms of “pure basic research” [7] that does not have to imply any practical applicability, but contributes to the (further) scientific understanding of a phenomenon or to the refinement of scientific methods. In contrast, they conceptualize *Application* as “pure applied research” that aims at increasing the applicability of research results in practice or at establishing policy decisions, i.e. implementation or translation of research [7]. We decided to take this differentiation as a model for our own analysis regarding the value of bioethical research.

Methodologically, these two value dimensions served as main deductive *categories* for the subsequent data extraction and analysis. This means a text passage was only included if it contained something explicitly or had, at least, to imply clearly something about the theoretical or practical value of the research. Typical examples for explicit and implicit statements can be seen in Table 2. Regarding explicit statements, we often used “*in vivo*” codes when analyzing the material, i.e. we adopted the original wording using only slight paraphrasing, while reformulating and heavily paraphrasing the content of the more implicit statements.

**Table 2:** Examples of explicit and implicit statements.

**Explicit** statements	“This study provides empirical evidence that can help policy makers in developing end-of-life regulations”^a^
	“Our discussion demonstrates the need for the various ethical issues to be considered and addressed in any decision to mandate cancer warning labels”^b^
**Implicit** statements	“We defend the permissibility of ritual male infant circumcision both ethically and from a human rights perspective”^c^
	“As a final note, I will build my argument on top of the premise established by other philosophers that biomedical enhancements are not in themselves morally problematic or wrong, simply in virtue of their being biomedical”^d^

^a^ Raijmakers et al. 2015, 149 [59]

^b^ Louise et al. 2015, 3 [68]

^c^ Jacobs and Arora 2015, 30 [66]

^d^ Nam 2015, 127 [37]

Two authors (MM and SS) read the full texts of the 40 articles to extract the data, and identified text passages within these articles which contained statements about the value of the research reported. They started independently with a tentative analysis and comparison of a subsample of eight articles (two articles per journal) in order to test the method and general approach, and to develop a shared understanding of how the two categories of *Advancing Knowledge* and *Application* can be conceptualized regarding bioethical research. After reconciling the interpretations of each text passage and the initial coding of these passages, the main analysis started.

### Data analysis and category building

As part of the main data analysis, MM and SS first coded the same 25% of the sample (equaling ten articles) and developed a list with definitions and examples of the codes independently. This procedure followed the principles of qualitative content analysis [73], including a deductive application of the two main categories and an inductive category building for fine-grained categories of the codes based on the text passages analyzed. However, the inductive category building finally followed a mixed strategy, as it was informed by some theoretical (“deductive”) reflections about what constitutes “valuableness” in bioethical research in analogy to biomedical research, i.e. the two main value dimensions.

The code lists of both coders were then merged through a comprehensive critical exchange of the respective understanding, also regarding the original text passages extracted that were anchoring the codes. Subsequently, the remaining 30 articles were coded independently based on the list of codes developed jointly and compared repeatedly after completion of the next eight to ten articles. The goal of the analysis was to reach a theoretical saturation with respect to our project’s research question. Regular team discussions took place on the application of the codes, the further development of the code list and the categories that could be built on this basis to enhance intercoder reliability.

The categories aimed at capturing value dimensions, corresponding to the two a priori main categories of *Advancing Knowledge* and *Application*. The category building was performed concurrently with coding, with new codes generating inductively new possible categories. After comprehensive discussions about the categories between MM and SS, and after a critical review by TF as a third author, some categories were merged, some codes redefined as being just examples for a specific category, and some categories eliminated as being “too far off” for our research question or as being redundant. Some categories also became first order categories for other categories, or new categories were introduced as first order categories to subsume hierarchically other, more concrete categories. These were rearranged as second or third order categories. The final categories were then streamlined by adjusting the wording to a consistent style.

## Results

**Fig 1:**
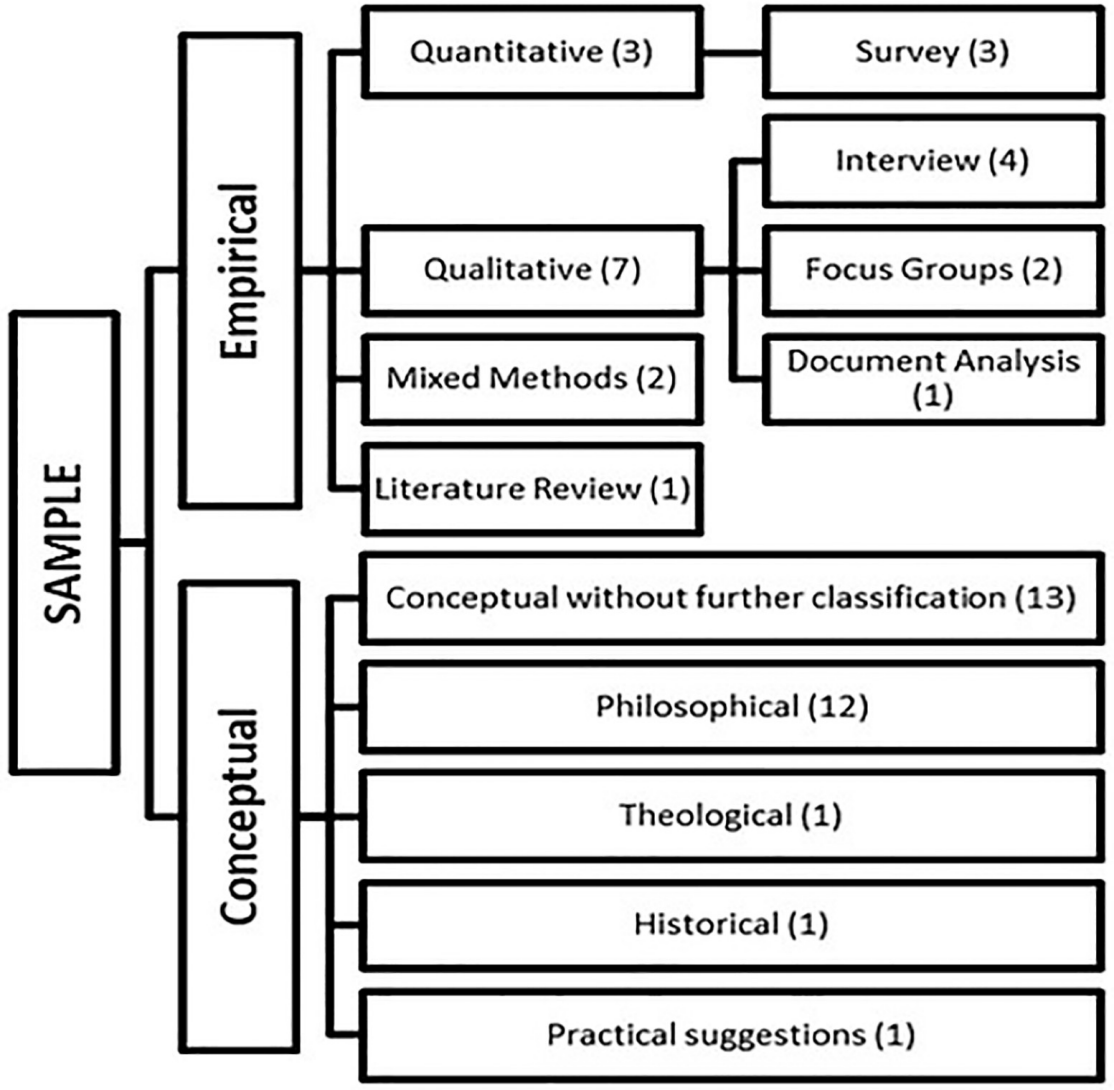
Distribution of article type.

40 articles published in 2015 in four major bioethics journals were subject to analysis. 12 articles were considered to be empirical studies, 28 articles conceptual papers; an overview of the different types of articles, as classified by the raters, can be found in Fig 1.

The qualitative data analysis resulted in a comprehensive coding system with a first basic division between the main dimensions of *Advancing Knowledge* and *Application*. Of the 62 total categories, 45 related to *Advancing Knowledge* and 17 related to *Application*. In the 40 articles, the range of author statements (text passages) that were coded using the 62 categories was 1 to 6 (i.e. up to six author statements were coded in an article); no article was without an author statement that was coded. 21 articles did not mention any dimension of *Application*; their value was restricted to *Advancing Knowledge*. The remaining 19 articles mentioned both—thus, no article only mentioned *Application*. The richness and complexity of the *Advancing Knowledge* part of the coding system was, thus, considerably higher compared to the application dimension.

The range of categories which were developed as result from the qualitative literature analysis can only be presented in an exemplary form in this paper (cf. S1 Fig for an extended “mindmap”-view). The thematic diversity of the categories is considerable and mirrors the heterogeneous character of bioethical research projects. As ethical questions potentially arise in all branches of biomedicine, the aims of concrete bioethical research projects vary widely with respect to targeted fields (e.g. research, clinical practice, health policy etc.) as well as with respect to the concreteness of their impact. The selection for data presentation in this publication has been made with the intent to provide as broad an overview as possible of the value dimensions derived from bioethical publications without losing theoretical depth.

### Advancing knowledge

The dimension of *Advancing Knowledge* is, at a first level, structured into 13 categories with the following titles:

- Adding relevancy to a topic
- Informing about societal support for current ethical/legal ideas or norms
- Identifying ethical problems
- Developing a better understanding of a moral phenomenon
- Applying ethical theory of ethical concepts
- Developing ethical theory
- Normative discussion of an ethical issue
- Assessing legal norms
- Adding a new topic to an ethical debate
- Opening/Intensifying debate on an adequate way of how to deal with an ethical problem
- Informing about structures, organizations and codes
- Addressing research issues
- Testing moral intuition

These categories represent the main ways in which the articles analyzed claim to contribute to a better knowledge of bioethical issues. They range from aspects which stand at the beginning of dealing with an ethical topic (“Adding relevancy to a topic,” “Identifying ethical problems”) to issues related to ethical theories (“Applying an ethical theory of ethical concepts,” “Developing ethical theory”), and include practice-related issues, such as the assessment of legal norms or the presentation and discussion of structures, organizations and professional codices.

**Fig 2:**
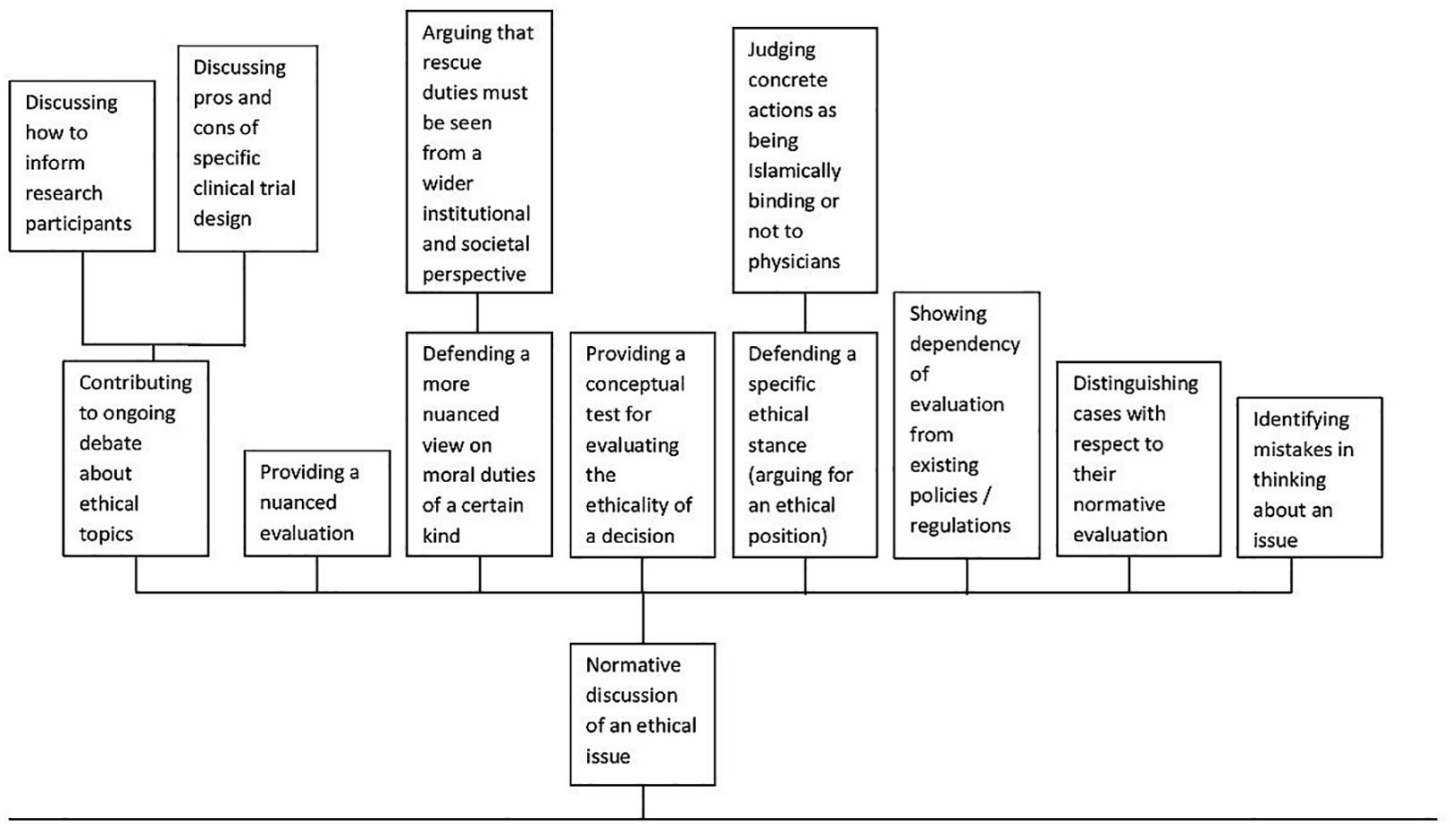
Code “Normative discussion of an ethical issue”.

The code “Normative discussion of an ethical issue” is, for example, further spread into sub-categories, such as “Defending a specific ethical stance” or “Providing a conceptual test for evaluating the ethicality of a decision” (see Fig 2). “Defending a specific ethical stance” refers to articles which argue for a decisive ethical position regarding a concrete issue, for example, a paper which exhibits aspects of using deception for involving children in research: “On this basis, we argue that non-planned deception requested by parents is very unlikely to be ethically acceptable”[61]. The code “Providing a conceptual test for evaluating the ethicality of a decision” was, for example, given to a paper which presents clear-cut criteria under which the authors judge a practice (ritual male infant circumcision) as being ethically unacceptable: “We offer a three-part test under which a parental decision might be considered an unacceptable violation of a child’s rights” [66].

**Fig 3:**
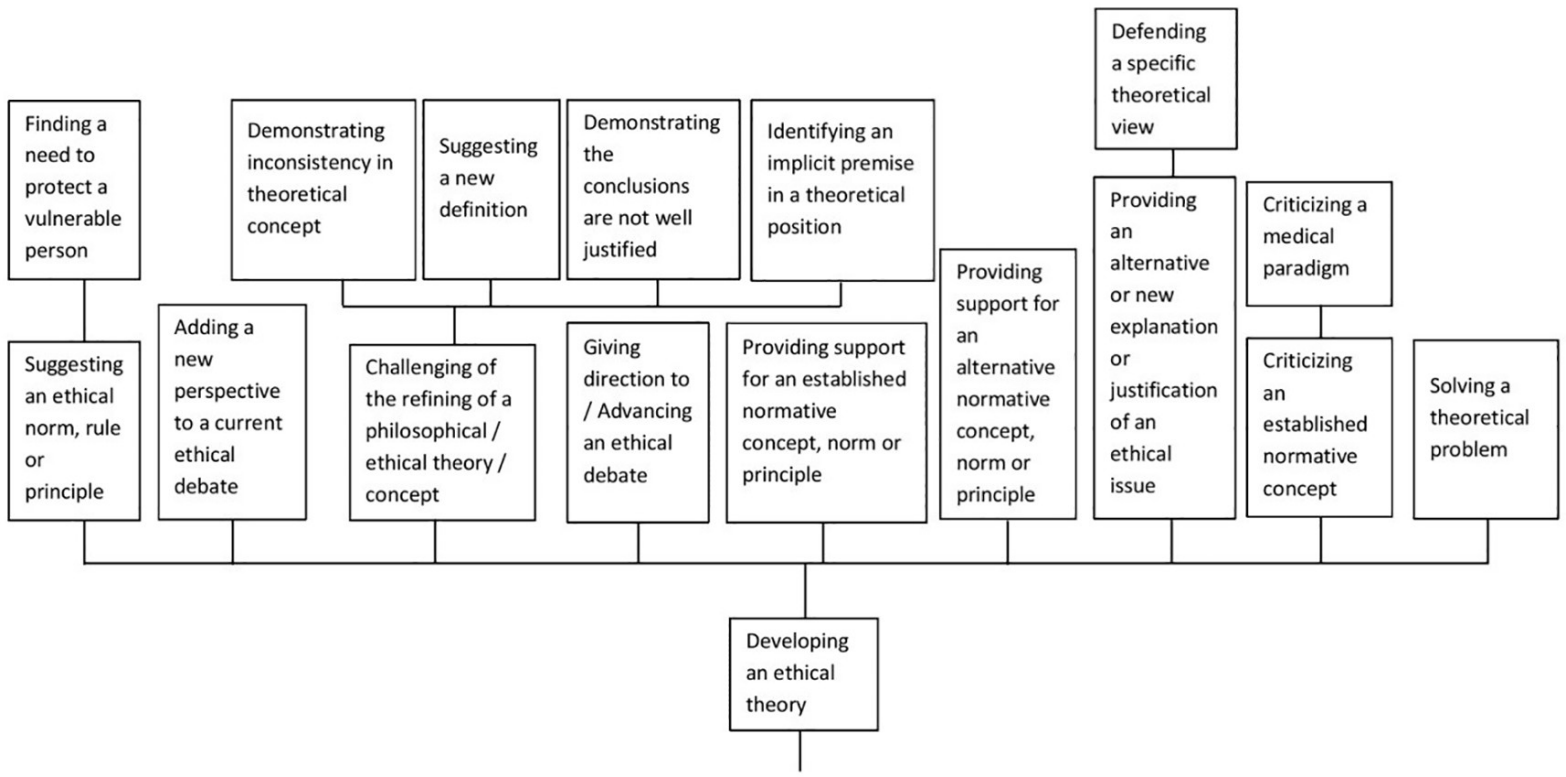
Code “Developing ethical theory”.

A second group of codes, still under the heading of *Advancing Knowledge* is related to the development of ethical theory and comprises such aspects as suggesting and criticizing ethical norms, giving direction to an ethical debate and solving theoretical problems (see Fig 3).

The code of “Giving direction to/advancing an ethical debate” has been assigned to articles which strive to frame the ethical discussion on a concrete issue in a distinct and innovative way, for example, concretely suggesting a new empirical approach to the subject or making attempts to advance the discourse between various stakeholders. The aim of giving direction can, however, also be addressed in a conceptual way, for example, when the authors declare that: “The purpose has been to bring forth distinctions and concepts that we believe can advance the debate about luck egalitarianism in this context” [54].

The development of ethical theory as a possible value of bioethical contributions can also consist of the solving of a theoretical problem. One example of this contribution is an article which discusses a paradox occurring regarding synthetic biology, namely, that the application of the precautionary principle can result in a situation where each way of action may result in catastrophic consequences. The authors declare that they present arguments which resolve this theoretical problem as a main contribution of their article [42].

### Application

The second major part of the coding system contains those aspects which are related to an immediate influence on health care, on the political or institutional practice of dealing with ethically contentious issues. The first level codes contained in this section are:

- Supporting ethical decision-making
- Supporting policy making
- Contributing to the development of ethics education, training and support
- Identifying needs
- Raising awareness
- Addressing recommendations
- Strengthening motivation and dedication in a profession
- Addressing political institutions

**Fig 4:**
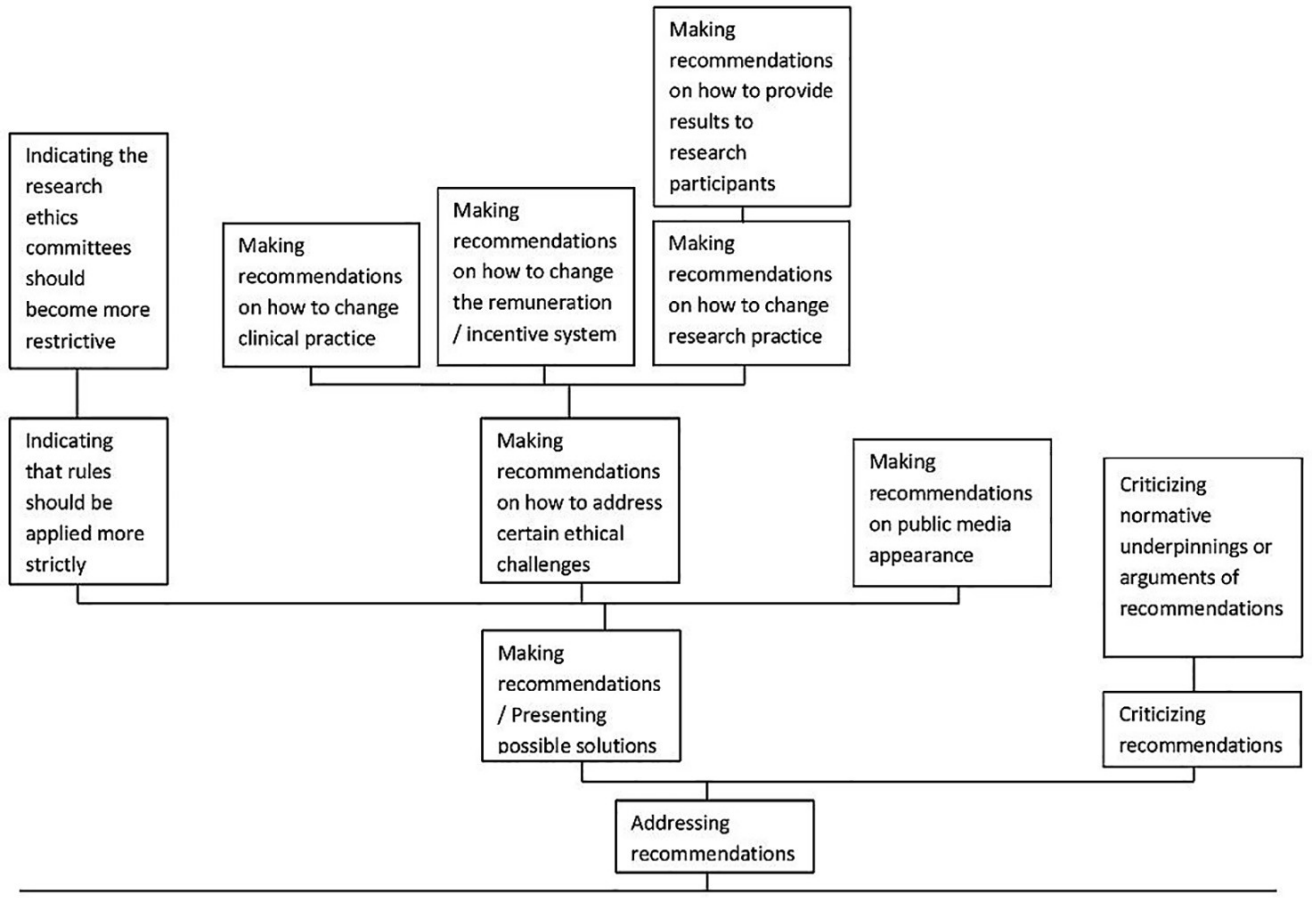
Code “Addressing recommendations”.

Methodologically, we decided to subsume article sections dealing with research ethics topics not under “Application,” but under *Advancing Knowledge*. One major section of the coding system on “Application” was related to the different dimensions of ethical recommendations which were addressed in the publications (see Fig 4).

Most codes were assigned to articles which declare that they make suggestions or present possible solutions to ethically controversial topics. The suggestions extended to such different fields as clinical practice, research practice or the health care remuneration system. Recommendations on changing clinical practice were, for example, made in an article dealing with conscientious objection towards performing abortions. The author suggests that: “As in other countries, GPs could be involved in early term abortions” [58]. Addressing recommendations could, however, also mean that authors plea that rules existing already should be followed more strictly, for example, in an article on placebo-controlled clinical trials which argues that “RECs should approve studies only when sufficient information has been provided for a solid judgement about the acceptability or undesirability of a placebo control” [46]. Addressing recommendations, however, could also mean criticizing the content of documents such as guidelines, for example, with respect to their underlying normative assumptions. A study on the social determinants of health does so regarding an integrative view on different policies: “We argue that the normative underpinnings of the HESC model are not sufficiently supported and that the policy recommendations do not necessarily follow from the arguments provided and may be inconsistent” [70].

## Discussion

### Advancing knowledge

Chalmers et al. understand “advancing knowledge” mainly in terms of “pure basic research” ([7], 156). In this respect, “advancing knowledge” corresponds with the idea of *scientific value*: Research and research results that, though not necessarily generating social benefit, are relevant for science itself and for further research. “Advancing knowledge,” understood in this way, means that our scientific explanations and predictions, our (conceptual) systematization and our understanding of the world around us is augmented or extended, even if this does not necessarily lead to altering the world (i.e. changing social practices).

The presence of this type of research, whose value has an effect particularly within the scientific system itself, is also mirrored in our study results on the self-declared value of bioethical publications. According to the data analysis, advancing knowledge in bioethics is mainly related either to a fuller understanding or appreciation of moral phenomena (e.g. codes “adding relevancy to a topic,” “identifying ethical problems,” or “normative discussion of an ethical issue”) or to the further development of ethical theory or ethical methodology. Thus, advancing bioethical knowledge has a double character: It is either descriptively or normatively related to a concrete ethical topic, which may (ultimately) lead to an ethical judgment; for example, about ethically contentious practices, structures or technologies. Or it is related to bioethical “tools” in the form of theories, concepts or methods.

According to these findings, the analogy of “advancing bio*medical* knowledge” to “advancing bio*ethical* knowledge” is, however, not unproblematic. In bioethics there is a distinction between “pure basic research” (e.g. philosophical inquiries into fundamental moral theories and concepts) and “applied ethics research,” which utilizes general moral theories for the discussion of concrete moral challenges or issues. However, two limitations arise in the analogy to biomedical research: Firstly, in practice, “applied ethics research” often has the character of “basic research,” even though in a more focused way. Secondly, in ethics some kind of “application” is always implied, as ethics—being a mainly normative discipline—deals basically with the question: “What shall we do?”. Ethics research, therefore, does not aim primarily for explanations or predictions, but for finding out what would be “the right thing to do,” a question which is always related to practical relevance. The qualitative analysis of bioethical publications, however, highlights how the specific value of bioethics in advancing knowledge lies either on the generic or on the case level.

The peculiarities of bioethics as academic discipline as arising from the text analysis also shed light on the question of scientific value in this field of research. When “science” is understood in strict terms of natural science or social science, one could even deny that bioethics provides any scientific value at all. If we understand “science” more broadly, however, also scholarly research from the humanities has to be included. As long as we then accept that scholarly work in the humanities can be research (i.e. is not something akin to art, or just a personal hobby), bioethical research—which uses philosophical, theological, historical and other methods of humanities—can have scientific (scholarly) value. This is illustrated by the results of our study that are part of the advancing knowledge dimension, e.g. in codes such as “Developing a better understanding of a moral phenomenon”, “Developing ethical theory”, “Normative discussion of an ethical issue” or “Adding a new topic to an ethical debate”. More generally spoken, scientific value here means contributing to an ongoing scholarly debate by providing new insights, questions, arguments, analyses of concepts, theories about bioethical topics etc.

The expectation towards bioethics to provide *some* scientific value to others must be seen as uncontroversial, as research is a collective process that is characterized by the exchange and dissemination of results, so that other researchers can uptake, react to and expand on them. Also “directed” research funded or conducted by private organizations might have some scientific value, as the results—when openly published—provide a contribution in the way of arguments (and counter-arguments) or a conceptual elaboration of a specific (political/ethical) position. However, just “thinking and writing for oneself” on bioethical topics would not count as “research”, but would indeed more be akin to a personal hobby. Therefore, contributing to bioethical research is necessarily related to the attempt of generating scientific value—which is also current practice as part of peer review or editorial processes when submitting a bioethics paper.

### Application

Regarding biomedical research, Chalmers et al. conceptualize “application” as “pure applied research” that aims at increasing applicability of research results in practice or at supporting the establishment of policy decisions ([7], 156). Research results are, therefore, not “an end in themselves,” as they might be in “pure basic research,” but have instrumental value in promoting something that is socially desirable, thus also conceptualized as the *social value* of (clinical) research ([74], 127).

Research which falls under the category of application thus is thought to have actual consequences for practice—at least conceivably—in the short-term. However, the impact of *bioethical research* and scholarship often appears rather in the long-term, and as a consequence of several research projects, not of a single piece of research [17]. Regarding the concrete impact, a further difference can be observed between biomedical and bioethical research: Biomedical research contributes to the further development of diagnostic, therapeutic or preventive measures and optimizes patient care, which is usually an ethically uncontested aim. Bioethics, by contrast, often comes into play when the goals or means of medical practice are doubtful, for example, when situations of moral distress arise in clinical practice or when political institutions have to decide about ethically contentious issues. The support which can be delivered by bioethical scholarship in such situations often does not consist of giving clear advice on action, but in the provision of strategies, methods and techniques which should enable fair and balanced ethical decision-making (cf. Mathews and colleagues who characterize the translation of bioethical research as “output into changes in thinking, practice, and policy” ([17], 66)). The multidirectional character of the application dimension (in contrast to “advancing knowledge”) is illustrated by the qualitative data analysis in the present study, which shows that this value dimension of bioethical research extends to such different fields as politics and policy making, clinical and research practice, clinical-ethical education and training, societal awareness and education in general (see e.g. codes such as “Making recommendations on how to address ethical challenges” or “Criticizing normative underpinnings or arguments of recommendations”).

Beyond such concrete examples it can still be discussed, however, whether bioethical research generates social value (comparable to biomedical research), and whether it should be required to do so. The reason for this is twofold. Firstly, social value is—compared to scientific value—a more contested concept concerning its meaning as well as its scope, even in clinical research (see e.g. [31,75,76]). Secondly, and related to the first point, applying the requirement of social value specifically to bioethical research seems more difficult than applying the requirement of scientific value.

In the clinical field, social value is, for instance, defined as “improvements in health” ([74], 127) for various potential beneficiaries (e.g. participants at a trial as direct beneficiaries vs. other patients that will profit from the intervention in future as indirect beneficiaries etc.). However, as Habets et al. discuss [75], social value is sometimes also extended to the general knowledge gain that can be useful in future for improving health, e.g. by providing a better understanding of a disease. Habets et al. do not approve this conceptual broadening as it makes it more difficult to pinpoint social value. Instead they propose to restrict social value as a value for society in terms of (anticipated) improvements on the wellbeing of patients, and not to knowledge gains in general.

Such an understanding of social value that is restricted to the improvement of wellbeing could, however, be too narrow for bio*ethical* research. Bioethical research does not typically lead to direct improvements of the wellbeing of patients, especially when “wellbeing” is understood physically, e.g. in terms of curing a disease or alleviating symptoms. Transferring the understanding of social value to bioethical research may thus work better if “wellbeing” is extended also to psychological and existential dimensions (e.g. being more at ease with a clinical intervention, feeling more respected, or being more involved in decision-making processes etc.). Looking at the list of possible value of bioethics as provided by Hofmann and Magelssen [18] or at the results of the application dimension of the present study reveals how such kinds of practical improvements can be subsumed under “social value”–such as improving policy, providing help for decision-making, criticizing current laws, sensitize practitioners to certain ethical issues etc.

While the characterization of social value might thus not be a general problem for bioethical research, giving a rationale for *requiring* bioethical research to have social value is much harder than it is for biomedical and especially clinical research. Whereas in *clinical* research risk-benefit (or “risk-value” [75]) assessments are a key issue, *bioethical* research is missing a proper correlate, apart from perhaps some empirical-ethical research settings where (I) there are human participants at all, and (II) they could be harmed in some way by participating [19]. And although bioethical research can certainly discuss and probably promote social justice [74]a general requirement that bioethical research itself has to be socially just cannot be plausibly argued for as there is often no harm or direct health benefit involved. So, whereas in clinical research, social value (as one facet of the application dimension) can be required for ethically legitimating research, it is unclear how this could hold true for bioethical research.

However, there is another argument for the possible requirement of providing (also) social value in bioethics. As e.g. Rogers argues [77], in bioethics, “[e]ven primarily empirical or descriptive scholarship has the underlying aim of contributing to some normative claim” ([77], 3), so it is impossible to conceptualize bioethics completely without at least *some* advocacy or even activism regarding the topics at hand (see also [78]). Even though it is debatable how much advocacy or activism is required—which also Rogers concedes–, the explicit or at least underlying ethical normativity and vicinity to practice makes a case for the possibility of generating social value (e.g. by contributing to ethical improvement of current practices) as well for the requirement that at least some bioethical research should also aim to have potential social value, i.e. has to fulfill a social value requirement as an ethical obligation.

Furthermore, when a bioethicist is paid for her/his research via public funds this is also related to the concomitant duty to publish and provide some benefit for the society. However, the extent to which bioethical research is publicly funded depends on political and societal circumstances, with countries funding vast institutions, chairs or specific projects in bioethics (as e.g. is currently the case in most European countries), and countries where bioethics research is not well covered by public resources but rather dominated by private and institutional initiatives (e.g. foundations or churches). The demand for social value might be weaker when there is private funding for bioethics research. In extremis, the funder could be an organization that aims for a “legitimation paper” serving their specific political purposes or advocacy. If we, however, consider research as an open and unbiased endeavor which must principally be open towards new and unexpected results, the requirement of providing social value holds for both, publicly and privately funded research.

In summary, bioethical research serves a genuine function with respect to its application dimension. It also has various target groups, such as health care professionals, patients, relatives, health care managers, policy makers and the general public (see also [17]), whereas biomedical research primarily addresses patients and health care providers. This divergent focus should be considered when judging the social value of bioethical research as emerging from the qualitative analysis of researchers’ statements.

### Relationship between “advancing knowledge” and “application”

In summary, both dimensions analyzed in the publications serve the aim of “making the world better” as an inherent goal of bioethical research: Without elaborated ethical knowledge, an appropriate basis for determining the best solution regarding ethically contentious questions is missing. Without the orientation towards application, however, no one profits from bioethical research results, and the benefit of this enterprise for individuals and society becomes doubtful. The qualitative data analysis revealed that a considerable proportion of publications do not address the application dimension and that the quantity and complexity of codes within the dimension of “advancing knowledge” is generally higher. If we regard the two dimensions as being inherently related with respect to bioethics, the application dimension often missing can be seen as a shortcoming in bioethical publications. In addition, both dimensions may come into conflict regarding policy making and the role of ethics experts in interdisciplinary commissions and councils. The relationship between “pure” ethical analysis and more practice-oriented approaches is, thus, context-dependent and has to account for the wide spectrum of tasks which can potentially be expected from bioethical scholarship.

### Limitations

Study limitations arise from the sampling of bioethical publications. The sample size and the procedure for selecting journals as well of selecting articles in these journals do not allow generalizable findings. Selecting other journals and articles, respectively, would possibly have resulted in other findings, particularly concerning the proportion of value dimensions attributed to “advancing knowledge” or “application”. This is especially true if articles representing bioethical research from non-bioethical journals would have been considered (e.g. *JAMA*, *NEJM*, *BMJ*, *Lancet*, *Science*, *Nature* etc.). Although generalizability does not constitute the aim of qualitative research, the scope of the results must nonetheless be interpreted with care. Further limitations accrue from the data analysis. Category-building and identifying text passages in articles both rely on interpretative tasks, especially regarding implicit statements. We tried to enhance the intercoder reliability of our analysis by consensus procedures between the three researchers. However, other researchers might have built different categories and would have assigned text passages to different categories. Nevertheless, as our study did not intend to classify particular articles and their possible value, but to use the articles only for collecting different value dimensions, we do not think that this limitation bears too much weight, i.e. it would not vastly reduce the usefulness of our results if other researchers had categorized a value dimension in another subcategory or found another value dimension we missed.

A final limitation could be seen in using an empirical approach in general. Its findings can only provide a descriptive “inventory” of value dimensions, lacking the theoretical and normative justification of why a specific value dimension *should* be accepted as a legitimate value dimension in the first place. So, the findings might not be (necessarily) *normatively* adequate, albeit they are *empirically* adequate, i.e. the value dimensions are corresponding to what researchers actually deem valuable. In contrast, a theoretical approach for determining value dimensions, for example based on reflections on what goals bioethics should reach (e.g. “respectful and just public health”, “respectful and just clinical care”, “improve health and science policy” [17]) or what functions in society or health care it should fulfill (e.g. [4]), might be normatively more adequate, but may lack empirical adequacy. Even though we are not denying that further theoretical/normative work is required—especially also regarding the relationship between the value dimensions of “advancing knowledge” and “application”–, we regard empirical adequacy as an important starting point for thinking about legitimate value dimensions, and therefore would not consider the empirical approach as a problematic limitation in general.

## Conclusions

With respect to the self-application of our research approach the question arises what value *our* paper contributes concerning the future development of bioethical scholarship and practice. Regarding the “advancing knowledge” dimension, the paper presents one possible approach for determining—in a first step—value dimensions of bioethical research. Additionally, it gives a limited insight into how researchers understand the value of their research. It also addresses the “application” dimension, as it aims at encouraging self-reflection about the value of research in bioethics and provides first ideas regarding this value. It is, therefore, a contribution to meta-research in bioethics, following the example of meta-research in biomedicine [79].

One upshot of our paper is that, irrespective of the factual reasons bioethical researchers have to research and publish (e.g. in the context of a PhD thesis, or agendas of research funding agencies and general societal and scientific trends), bioethical researchers should nonetheless reflect more upon the scientific and/or social value of their research and publications. This implies that they should not only address this question more prominently in their publications or other forms of research presentation, but should also try to align their research agendas, secondary interests of research (e.g. academic career prospects) and focus of their publications (more for the “esoteric” debate within the field, or more with “translational” prospects for other fields or clinical practice) regarding the expected scientific and especially social value.

Further investments in meta-research on bioethics could include empirical work. In addition to the present study bioethicists’ perspectives on the value of their own work, for example, could be elicited in an explorative qualitative study followed by a survey of the scientific community. In combination with our study such research could build the basis for a development of more concrete criteria or “assessment tools” to estimate the value of particular bioethical papers or research projects, adjusted to different types of bioethical research [80], as also proposed in a comparable way by Mathews and colleagues [17]. In the long run, this could even lead to a “Harm-Benefit-Assessment” for bioethical research that could be set as a research ethics standard, just as Risk-Benefit-Assessment is a standard for preclinical and clinical trials. Such a value assessment could be included in reporting guidelines for bioethical publications. The present study has already revealed some of the possible dimensions that could be addressed in such a guideline.

Finally, the evaluation of the impact of bioethical research could be improved: Does bioethical research change social practice in biomedicine and health care, and, if yes, is it in an ethically desirable way? What happens if ethical norms or “tools” are implemented in a practice? And how could implementation be improved (cf. [4])? We think that this kind of meta-research is still widely lacking in bioethics. However, it would certainly increase or, more optimistically, even demonstrate the relevance of our field. It would also give empirical feedback on the question about what actually makes bioethics valuable.

## Supporting information

S1 Fig “Mindmap” of all categories. (TIF)
