# Supplementary figures and images for: The value of bioethical research: A qualitative literature analysis of researchers’ statements
Source: PLoS One. 2019 Jul 29;14(7):e0220438. doi: 10.1371/journal.pone.0220438 (PMC6663028; doi:10.1371/journal.pone.0220438)

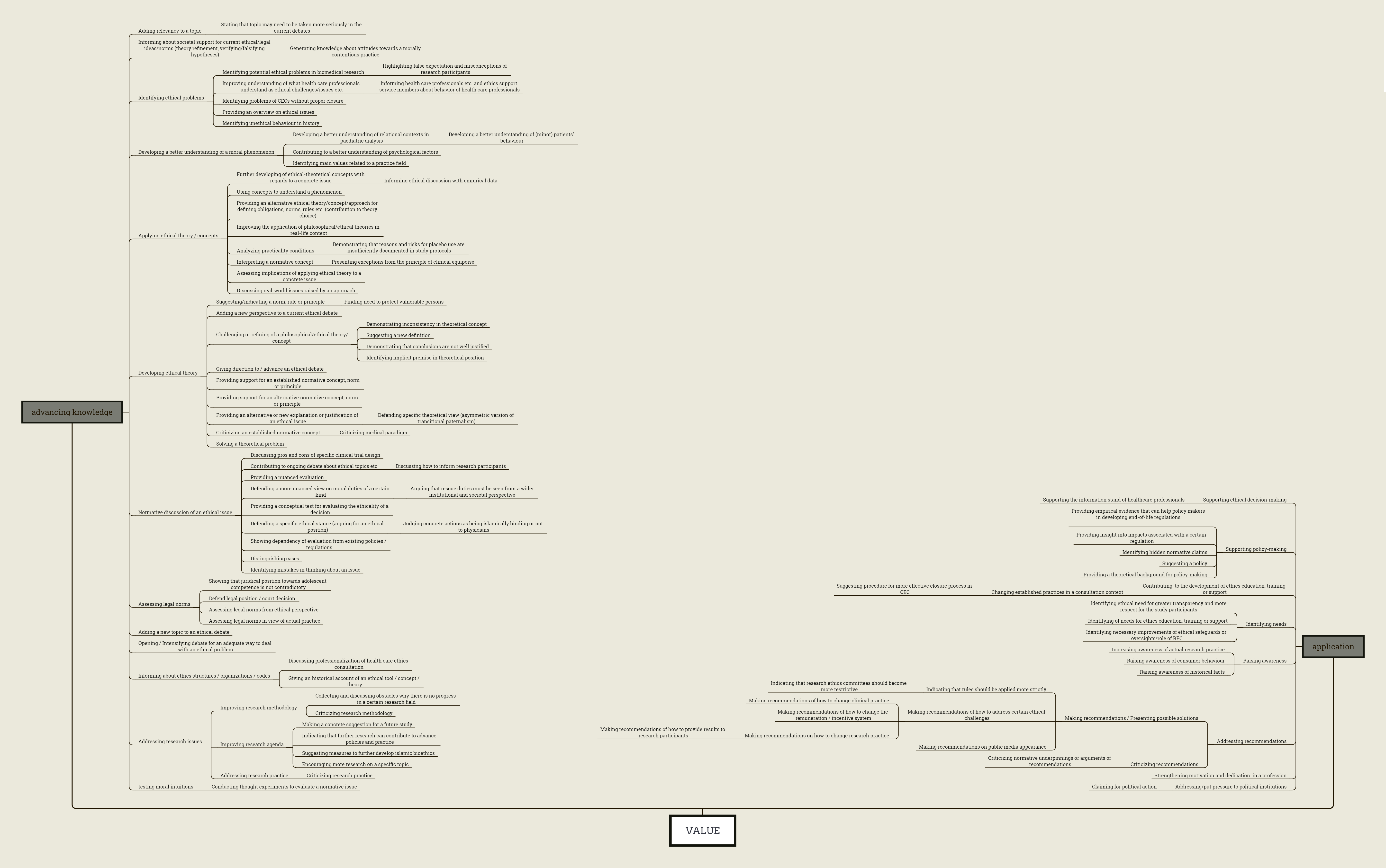

Supplement: S1 Fig — (TIF) [file pone.0220438.s001.tif]
